# Metagenomic and Pathogenic Assessments Identify a Pathogenic Porcine Reproductive and Respiratory Syndrome Virus 1 with New Deletions from Adult Slaughter Pig in 2022

**DOI:** 10.1155/2023/1975039

**Published:** 2023-04-13

**Authors:** Chen Li, Ming Qiu, Shubin Li, Zhe Sun, Zitao Huang, Wenhao Qi, Yuejia Qiu, Jixiang Li, Binghui Feng, Dashi Zhao, Hong Lin, Wanglong Zheng, Xiuling Yu, Kegong Tian, Kewei Fan, Jianzhong Zhu, Nanhua Chen

**Affiliations:** ^1^College of Veterinary Medicine, Yangzhou University, Yangzhou 225009, China; ^2^National Research Center for Veterinary Medicine, Luoyang 471000, China; ^3^Animal Health Supervision Institute of Fengxi District, Chaozhou 521031, China; ^4^Longyan University and Fujian Provincial Key Laboratory for Prevention and Control of Animal Infectious Diseases and Biotechnology, Longyan 364012, China; ^5^Joint International Research Laboratory of Agriculture and Agri-Product Safety, Yangzhou 225009, China; ^6^International Research Laboratory of Prevention and Control of Important Animal Infectious Diseases and Zoonotic Diseases of Jiangsu Higher Education Institutions, Yangzhou 225009, China; ^7^Comparative Medicine Research Institute, Yangzhou University, Yangzhou 225009, China; ^8^Jiangsu Co-Innovation Center for Prevention and Control of Important Animal Infectious Diseases and Zoonoses, Yangzhou University, Yangzhou 225009, China; ^9^Key Laboratory of Animal Pathogen Infection and Immunology of Fujian Province, Fuzhou 350002, China

## Abstract

Since we first reported porcine reproductive and respiratory syndrome virus 1 (PRRSV1) wild type strains in mainland China in 2011, PRRSV1 infection has been detected in more than 20 provinces in China. During the routine investigation of PRRSV1 epidemiology in 2022, we isolated a novel PRRSV1 strain (SD1291) from an adult slaughter pig in Linyi, Shandong Province. The SD1291 could only be isolated with primary alveolar macrophages (PAMs), not with Marc-145 cells. In addition, the 2022 SD1291 isolate has higher *in vitro* replication efficacy than the 2014 PRRSV1 HLJB1 isolate in PAMs. Due to high genetic variation, the complete genome of SD1291 was determined by metagenomic sequencing, which showed that SD1291 shares the highest genome similarity (88.12%) with the PRRSV1 HeB47 isolate. Sequence alignment results identified a four-amino-acid deletion in nsp2 and a five-amino-acid deletion in the GP3 and GP4 overlap region of SD1291. A complete-genome-based phylogenetic tree showed that SD1291 is grouped with BJEU06-1-like PRRSV1 isolates. A piglets' challenge study showed that SD1291 can cause high fever (the highest is 41°C), reduced weight gain, mild lung consolidation, and interstitial pneumonia indicating that SD1291 is a pathogenic PRRSV1 isolate. Overall, this study first identified a novel pathogenic PRRSV1 isolate from an adult slaughter pig in China. Our findings also suggested that new PRRSV1 variants could escape the current PRRSV vaccination system and circulate in adult swine herds, which deserve more attention.

## 1. Introduction

Porcine reproductive and respiratory syndrome (PRRS) was first reported in North America in 1987 and then in Western Europe in 1990 [1, 2]. The etiological agent of PRRS, PRRS virus (PRRSV), was first isolated in the Netherlands in 1991 and subsequently in the United States [2, 3]. Due to antigenic and genetic differences, European and North American PRRSV isolates were previously divided into two genotypes and are currently divided into two species (PRRSV1 and PRRSV2) [4, 5]. Furthermore, PRRSV1 can be classified into three subtypes, while PRRSV2 is clustered within 9 lineages [6]. PRRSV2 is mainly prevalent in North America and Asia, while PRRSV1 is predominant in Europe. However, subtype 1 PRRSV1 has also spread to other countries in North America and Asia [7–10].

In China, PRRSV2 was first identified in 1995 [11]. In 2006, highly pathogenic PRRSV2 (HP-PRRSV2) emerged in China [12]. NADC30 was isolated in the United States in 2008, which is a moderately virulent PRRSV2 [13]. Since 2013, NADC30-like PRRSV2 has been prevalent in Chinese swine herds [14]. NADC34 is an ORF5 restriction fragment length polymorphism (RFLP) in the 1-7-4 lineage PRRSV2, which was isolated from the United States in 2014 [15]. From 2017, NADC34-like PRRSV2 isolates have become predominant in China [16]. Currently, HP-PRRSV2, NADC30-like PRRSV2 and NADC34-like PRRSV2 are predominant strains in China. Meanwhile, we first isolated wild-type PRRSV1 strains (BJEU06-1 and NMEU09-1) in mainland China from 2006 to 2009 [8]. Since then, increasing numbers of PRRSV1 strains have been detected in at least 20 provinces of China [8, 17–23]. In addition, recombinants between modified live vaccine (MLV) strains and wild-type PRRSV1 strains or between two MLV strains have also been found in China [18, 24]. Currently, all Chinese PRRSV1 isolates belong to subtype 1 and can be further divided into four subgroups (Amervac-like, NMEU09-1-like, HKEU16-like and BJEU06-1-like) [24].

PRRSV is a positive-sense, single-stranded RNA virus belonging to the *Arteriviridae* family within the *Nidovirales* order [25]. The PRRSV genome is about 15 kb in length, containing a 5′-capped and 3′-polyadenylated region, an untranslated region (UTR) at both 5′- and 3′-end, and at least ten open reading frames (ORFs) [26]. ORF1a and ORF1b encode two large nonstructural polyproteins, pp1a, and pp1ab, which can be processed into at least 16 nonstructural proteins (NSPs) [27]. ORF2 to ORF7 encode eight relatively small structural proteins, including four membrane associated glycoproteins (GP2a, GP3, GP4, and GP5), three unglycosylated membrane proteins (E, ORF5a and M) and a nucleocapsid protein (N) [28]. PRRSV nsp2 (also nsp2N) contains the hypervariable region, in which deletions and insertions are commonly observed [10, 28]. GP3 also contains a hypervariable region at the carboxy-terminal end overlapping with GP4, encoding another hypervariable protein, PRRSV [8, 29, 30].

Even though PRRSV1 has been detected in the majority of areas of China, the pathogenic characterization of novel PRRSV1 isolates is still very limited. Here we isolated a PRRSV1 strain from the lung of an adult slaughter pig in 2022. Due to high genetic variation, the complete genome of the 2022 PRRSV1 isolate was determined by metagenomics sequencing. Furthermore, the pathogenicity of the novel PRRSV1 isolate was also evaluated in piglets.

## 2. Materials and Methods

### 2.1. Clinical Sample Information

During the routine investigation of PRRSV1 epidemiology, a total of 257 lung samples were collected from slaughter pigs in eight cities (Linyi and Dezhou of Shandong province, Zhumadian, and Zhoukou of Henan province, and Neijiang of Sichuan province, Hangzhou of Zhejiang province, Chaozhou of Guangdong province, and Yangzhou of Jiangsu province) of China from February 24 to March 30 in 2022 (Table 1). A PRRSV1 real-time RT-PCR assay was used for PRRSV1 detection [31]. Briefly, total RNA was extracted from lung homogenate using the TRIpure reagent (Aidlab, China). The first-strand of cDNA was synthesized using PrimeScript 1^st^ Strand cDNA Synthesis Kit (TaKaRa, Japan). PRRSV1 specific probe and primer pairs, the Premix Ex Taq (Probe qPCR, 2x) (TaKaRa, Japan) and StepOne Plus Real-Time PCR System (Thermo Fisher Scientific) were used for PRRSV1 real-time PCR detection.

### 2.2. Virus Isolation and Immunofluorescence Assay (IFA)

Virus isolation and IFA detection were performed in both Marc-145 cells and primary alveolar macrophages (PAMs) as previously described [32]. PAMs were prepared with lung lavage fluid from 6-week-old healthy piglets [33, 34]. PAMs were cultured in RPMI-1640 medium (HyClone) supplemented with 10% FBS (AllBio), 100 U/ml penicillin, and 100 *μ*g/ml streptomycin (Solarbio). Marc-145 cells were cultured in DMEM (HyClone) supplemented with 10% FBS [35]. The SD1291 lung homogenate was used for infection. The infected Marc-145 cells were monitored daily for cytopathic effects (CPE) for 7 days. The infected PAMs and Marc-145 cells were also collected at 72 hpi and used for IFA detection. Briefly, the infected cells were washed once with PBS and fixed using 4% paraformaldehyde. Cells were permeabilized with PBS containing 0.5% TritonX-100 for 10 min and blocked with PBS containing 1% BSA for 2h. PRRSV-specific murine mAb 15A1 (1 : 500) against N protein was used as the primary antibody ,while DyLight 594 goat antimouse IgG (1 : 1000, Invitrogen, USA) was used as the secondary antibody. Cellular nuclei were counterstained with 4′, 6-diamidino-2-phenylindole (DAPI). The results were evaluated with an IX53 inverted fluorescence microscope (Olympus, Japan).

### 2.3. Metagenomic Sequencing

Due to high genetic variation, the complete genome of thePRRSV1 SD1291 isolate could not be determined using 16 primer pairs as we previously described [8]. Therefore, total RNA from SD1291-infected PAMs was sent to Tanpu Biotechnology Co. Ltd. (Shanghai, China) for metagenomic sequencing as previously described [36]. In details, viral RNA was firstly extracted, fragmented, and then reverse transcribed into double-stranded cDNA and used for library preparation with the TruSeq™ DNA Sample Prep Kit (Illumina). Bridge PCR was executed with the TruSeq PE Cluster Kit (Illumina), and the Illumina NovaSeq 6000 PE150 sequencing was performed to produce raw data. Clean reads were generated by removing reads containing poly-N, reads containing adapters, and low-quality reads from raw data. In addition to host contamination, the ribosomal RNA and bacteria sequences were further filtered by comparison with the NCBI database using BBMap software. The *de novo* assembly and assembly evaluation were carried out using SPADES and SOAPdenovo software. The final scaffolds were subjected to viral sequence comparison in NCBI NT and viral RefSeq databases.

### 2.4. Sequence Alignment, Phylogenetic, and Recombination Analyses

To evaluate evolutionary relationships of the novel SD1291 isolate with other PRRSV strains, multiple sequence alignment was performed using ClustalX 2.0 based on 55 representative complete genomes (50 PRRSV1 and 5 PRRSV2). Phylogenetic trees were constructed based on the aligned complete genomes via MEGA 6.06 using the neighbor-joining method. The robustness of phylogenetic trees was evaluated by bootstrapping with 1000 replicates. To test the potential role of recombination in the generation of SD1291, multiple genome alignment was also submitted to screen potential crossover events by recombination detection program 4 (RDP4) [37] and SimPlot 3.5.1 [38]. To analyze the similarities among the SD1291 isolate and other PRRSV1 strains, the complete genome and each gene of the SD1291 isolate were also compared with five representative PRRSV1 strains (LV, Amervac, BJEU06-1, HeB47, and HLJB1) using DNAMAN (https://www.lynnon.com).

### 2.5. Animal Challenge Study

To evaluate the pathogenicity of the novel PRRSV1 SD1291 isolate, an animal challenge study was performed using 4-week-old PRRSV-free piglets. The piglet challenge study was approved by the Animal Welfare and Ethics Committee at the College of Veterinary Medicine of Yangzhou University with the reference number of 202209008. Ten piglets (a litter) were divided into two groups (five for each group). Piglets in the first group were nasally and intramuscularly inoculated with 2 mL of 10^5.0^ TCID 50/mL SD1291 isolate (3^rd^ passage), while piglets in the second group were inoculated with RPMI-1640 and set as a mock-infection group.

Clinical symptoms, rectal temperature, and body weight were monitored daily during the first two weeks after the challenge. The body weight was measured weekly, and serum samples were collected regularly for the analysis of viremia and antibody levels. The dynamics of viremia were tested by the PRRSV1 real-time RT-PCR assay [31]. PRRSV-specific antibodies in the sera were detected by the HerdCheck PRRS × 3 ELISA Kit (IDEXX, USA). The threshold for seroconversion was set at a sample-to-positive (s/p) ratio of 0.4. The pigs that survived until 21 days post challenge (DPCDPC) were euthanized, and tissue samples from each organ were collected for histopathological and immunohistochemical examinations as previously reported [39].

### 2.6. Statistical Analysis

The data of viremia, antibody level, rectal temperature, and body weight in this study were shown as means ± standard deviations (SD). The differences between groups were determined by a Mann−Whitney *U* test using Graphpad Prism 8 XML project [40]. A *p* value < 0.05 was considered to be statistically significant.

## 3. Results

### 3.1. PRRSV1 Was Detected in an Adult Slaughter Pig in China 2022

During the routine investigation of PRRSV1 epidemiology in 2022, a total of 257 lung samples were collected. Only one sample (sample number 1291 and named SD1291) from Linyi, a city in Shandong province, was PRRSV1 positive (Table 1). Retrospective analysis showed that the SD1291 sample was collected from an adult slaughter pig from a farmer's market. Other common swine viral pathogens, including PRRSV2, classical swine fever virus (CSFV), porcine epidemic diarrhea virus (PEDV), transmissible gastroenteritis virus (TGEV), African swine fever virus (ASFV), porcine parvoviruses (PPVs), and porcine Circoviruses (PCVs), were not detected in the SD1291 lung sample (data not shown).

### 3.2. PRRSV1 SD1291 Was Isolated in PAMs

**Figure 1:**
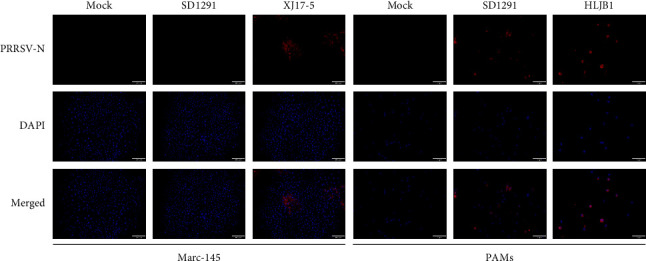
SD1291 was successfully isolated with PAMs but not with Marc-145 cells. Marc-145 cells and PAMs inoculated with SD1291 lung homogenate were examined by IFA at 72 hpi. PRRSV-specific antigen could be detected in SD1291 infected PAMs but not in SD1291 infected Marc-145 cells. HP-PRRSV2 XJ17-5 isolate was used as a positive control for Marc-145 cell infection and PRRSV1 HLJB1 isolate was used as a positive control for PAMs infection.

**Figure 2:**
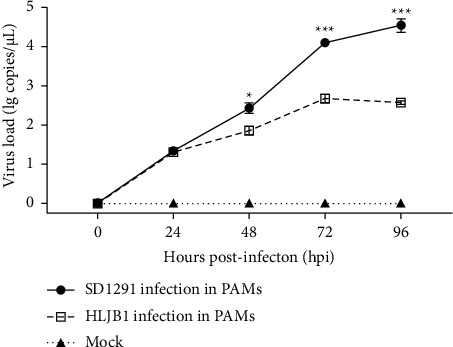
SD1291 isolate showed a higher in vitro replication efficacy than HLJB1 isolate in PAMs. Multiple steps grow curves showed that SD1291 has significantly higher virus load than HLJB1 from 48 hpi to 96 hpi.

To further characterize the 2022 PRRSV1 stain, the SD1291 lung homogenate was used for virus isolation with both Marc-145 cells and PAMs. The successful isolation of PRRSV1 was confirmed by IFA staining at 72 hpi. As shown in Figure 1, PRRSV-specific fluorescence could be observed only in SD1291 infected PAMs but not in SD1291 infected Mar-145 cells, indicating that SD1291 could not be isolated with Marc-145 cells but could be isolated with PAMs. Multiple step growth curves showed that the 2022 SD1291 isolate has higher *in vitro* replication efficacy than 2014 PRRSV1 HLJB1 isolate in PAMs (Figure 2). These results confirmed that SD1291 was successfully isolated in PAMs.

### 3.3. Metagenomc Sequencing Determines the Highly Mutated SD1291 Genome

**Figure 3:**
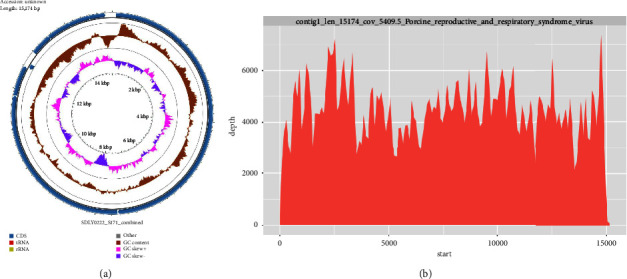
Metagenomic sequencing determined the PRRSV1 SD1291 genome. (a) Only a 15174 bp length sequence was obtained from the metagenomic sequencing. (b) The average coverage of the 15174 bp length sequence is 5409. The obtained sequence shared the highest similarity with PRRSV1 HeB47 isolate.

To analyze the molecular characteristics of the SD1291 isolate, the complete genome of SD1291 was determined by metagenomic sequencing. A total of 30.3 million 150 bp paired-end clean reads were generated for SD1291 isolate. Quality tests showed that the Q20 and Q30 rates were 96.95% and 92.46%, respectively, (Table S1). These results suggested the satisfactory quality of viral metagenomic sequencing. After the filtration of ribosomal RNA, host contamination, and bacteria sequences, 0.51 million reads (1.69%) were obtained and used for virus mapping. Only one 15174 bp length sequence was generated (Figure 3(a)). The average coverage for the entire sequence was 5409 (Figure 3(b)). BLAST results showed that the obtained sequence shares the highest similarity with the PRRSV1 HeB47 isolate (MN927228).

**Figure 4:**
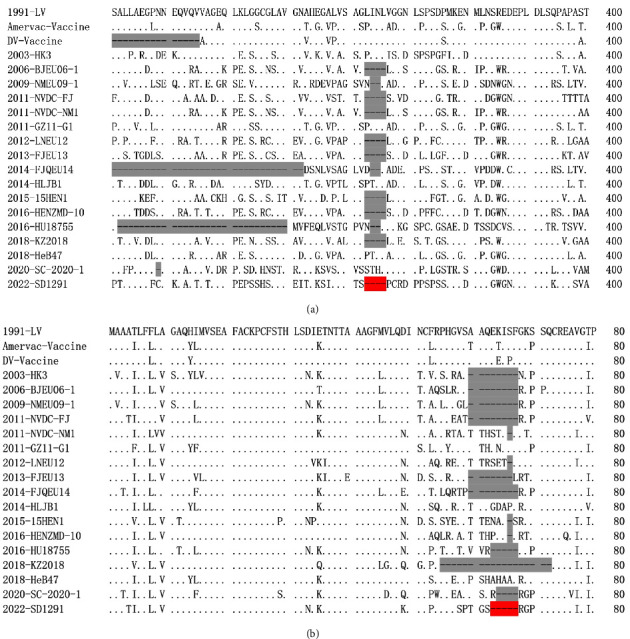
The amino acid alignments identified deletions in nsp2 and GP3-GP4 overlap region of the SD1291. (a) A four amino-acid deletion was identified in positive 363–366 of nsp2 of SD1291, which is identical to BJEU06-1, NVDC-FJ, NVDC-NM1, LNEU12, FJEU13, 15HEN1, HENZMD-10, and KZ2018 isolates. (b) A five amino-acid deletion was observed in positive 73–77 of GP4 overlapped with GP3, which is identical to HU18755 isolate. The two deletions of the SD1291 were highlighted in red and deletions in other PRRSV1 isolates were marked in grey.

**Figure 5:**
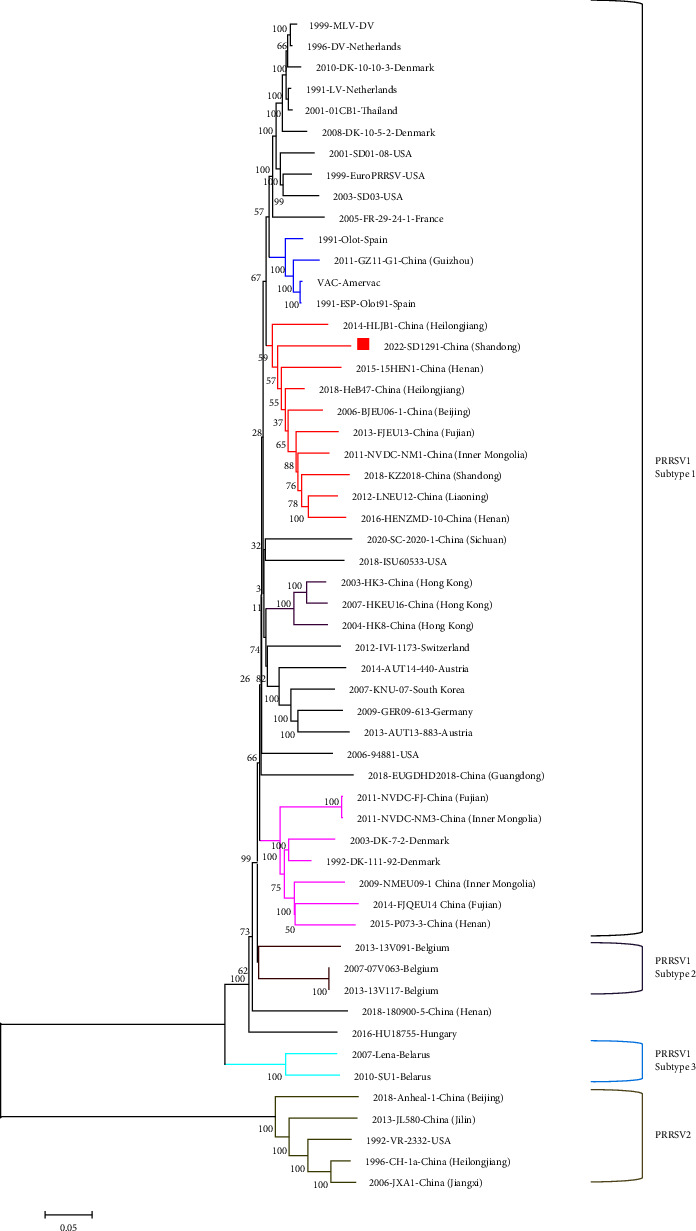
Genome-based phylogenetic analysis indicated that SD1291 is a BJEU06-1-like PRRSV1 isolate. Phylogenetic tree based on 50 PRRSV1 and 5 PRRSV2 genomes showed that SD1291 is clustered with BJEU06-1-like isolates, confirming that SD1291 is a novel PRRSV1 isolate. Each virus was showed by year of isolation, virus name and country (and province if isolated in China). Three subtypes of PRRSV1 and four subgroups of Chinese isolates in subtype 1 PRRSV1 were shown in different colors. The SD1291 isolate was marked with a red square.

Sequence alignment and comparison results showed that the complete genome of SD1291 is 15068 bp excluding the adapter sequence and 3′ Poly (A) tail. The SD1291 genome shares the highest genome similarity (88.12%) with PRRSV1 HeB47 isolate was followed by BJEU06-1 isolate (88.09%), prototypic PRRSV1 LV strain (87.59%), and Amervac vaccine strain (86.19%) and HLJB1 recombinant (85.28%). Each protein comparison showed that nsp2N is the most hypervariable nonstructural protein, while GP3 is the most hypervariable structural protein (Table 2). Furthermore, a four-amino-acid deletion was identified in nsp2 of SD1291 (Figure 4(a)). Meanwhile, a five-amino-acid deletion was observed in the GP3 and GP4 overlap region (Figure 4(b)). The four-amino-acid deletion in nsp2 of SD1291 is identical to BJEU06-1, NVDC-FJ, NVDC-NM1, LNEU12, FJEU13, 15HEN1, HENZMD-10, and KZ2018 isolates, while the five-amino-acid deletion in the GP3 and GP4 overlap region is identical to the HU18755 isolate. A complete-genome based phylogenetic tree showed that SD1291 was clustered with BJEU06-1-like PRRSV1 isolates (Figure 5). Recombination analyses by RDP4 and SimPlot softwares suggest that SD1291 is not a recombinant virus (data not shown). All these results indicated that SD1291 is a novel PRRSV1 isolate.

### 3.4. SD1291 Is a Pathogenic Isolate for Piglets

**Figure 6:**
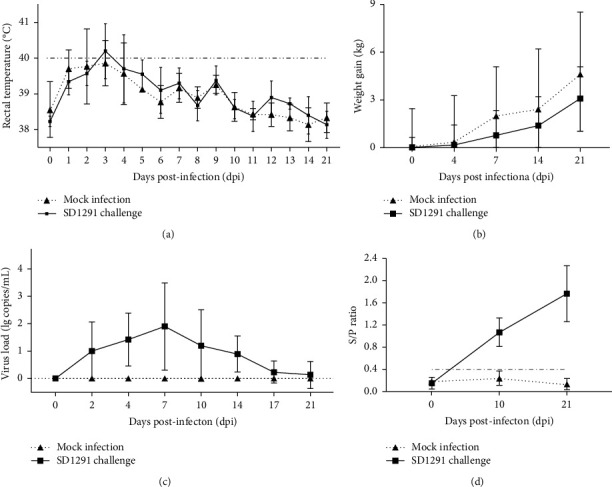
Dynamics of rectal temperature, weight gain, viremia and antibody level during inoculation and challenge studies. (a) SD1291 challenge could cause high fever (the highest of 41°C) from 2 DPC to 6 DPC. (b) SD1291 challenge could reduce the weight gain but with no significant difference (*p* > 0.05). (c) SD1291 challenge induced viremia from 2 DPC to 21 DPC. (d) SD1291 challenge could induce PRRSV-specific antibody response since 10 DPC.

**Figure 7:**
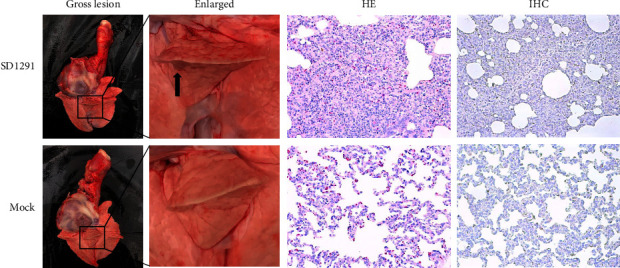
Lung gross lesion, histopathological, and immunohistochemical examinations. (Gross lesion) Mild lung consolidation was observed in the SD1291 challenged pigs but not in mock-infected pigs. (HE) Interstitial pneumonia with infiltration of lymphocytes and widened alveolar septum could be observed in the SD1291 challenged pigs but not in mock-infected pigs. (IHC) PRRSV-specific antigen was not detected in any pigs at 21 DPC.

To evaluate the pathogenicity of the 2022 PRRSV1 SD1291 isolate, a pig challenge study was performed. Clinical symptoms including coughing, anorexia, and diarrhea were observed in SD1291 challenged pigs but not in mock-infected pigs. Compared with normal rectal temperature in the mock-infected pigs, four of the five pigs inoculated with the 3^rd^ passage of SD1291 showed high fever (≥40°C) from 2 DPC, to 6 DPC with the highest at 41°C (Figure 6(a)). The body weight gain in SD1291-challenged pigs was lower than that in mock-infected pigs, but with no significant difference (*p* > 0.05) (Figure 6(b)). Viremia was first detected in one pig at 2 DPC and in all pigs at 4 DPC (Figure 6(c)), while PRRSV specific antibodies could be detected since 10 DPC in all SD1291-challenged pigs (Figure 6(d)). During necropsy examination, mild lung consolidation was observed in SD1291 challenged pigs but not in mock-infected pigs (Figure 7, gross lesion). Histopathological examination identified the widened alveolar septum and lymphocytes infiltration in SD1291 challenged pigs (Figure 7, HE). However, PRRSV antigen could not be found in all pigs at 21 DPC during the immunohistochemical examination (Figure 7, IHC). Overall, these results indicated that SD1291 is a pathogenic PRRSV1 isolate for piglets.

## 4. Discussion

PRRSV2 is still the predominant PRRSV isolate in China, however, the rate of PRRSV1 infection is gradually increasing in recent years. PRRSV1 has been detected in more than 20 provinces in China. A previous epidemiological investigation showed that the PRRSV1 positive rate was 24.8% (186/750) in 50 farms in Guangdong province [20]. Furthermore, the PRRSV1 SC2020-1 isolate is associated with a 15% abortion rate in sows [22]. These previous studies reminded us that more attention is required for PRRSV1 monitoring in China. In this study, we evaluated the prevalence of PRRSV1 in adult pigs and isolated a novel 2022 PRRSV1 strain (SD1291) from an adult slaughter pig. Metagenomic sequencing identified that SD1291 shared low similarity (≤88.12%) with current PRRSV1 isolates concurrently containing deletions in the nsp2 and GP3 and GP4 overlap regions. Pathogenic analysis showed that SD1291 is a pathogenic isolate for piglets. Therefore, this study first confirmed the circulation of pathogenic PRRSV1 isolates in China's adult pigs.

According to previous PRRSV1 epidemiological studies in China, the PRRSV1 positive rates could be significantly different. For instance, Liu et al. showed that only 0.26% (4/1566) of the samples collected from 2013 to 2014 in Fujian Province were PRRSV1 positive [17]. In addition, our previous study showed that only 0.28% (2/712) of the clinical samples collected from 2016 to 2019 were PRRSV1 positive [42]. The detection of 260 clinical samples collected from 2017-2018 in nine provinces showed that the PRRSV1 positive rate is 1.15% (3/260) [43]. In 2020, an epidemiological investigation in Henan Province showed that 10.61% (14/132) clinical samples were PRRSV1 positive [44]. Surprisingly, the epidemiological investigation in Guangdong province in 2016 using 750 clinical samples collected from diseased pigs with respiratory signs or reproductive disorders showed that the PRRSV1 positive rate is 24.8% (186/750) [20]. In this study, the PRRSV1 positive rate was 0.39% (1/257) in clinical lung samples, indicating that the PRRSV1 is sporadically prevalent in Chinese adult pigs. Overall, these studies suggested that the PRRSV1 positive rate could be affected by different detection regions, different age and health status of pig herds. The positive rate might dramatically increase in an outbreak situation.

Nsp2 and GP3 are hypervariable regions of PRRSV [26, 28–30]. Mutations, insertions, and deletions are commonly observed in these regions [10, 21, 29]. BJEU06-1, NVDC-FJ, NVDC-NM1, LNEU12, FJEU13, 15HEN1, HENZMD-10, and KZ2018 isolates have the same four-amino-acid deletion in nsp2 of SD1291, while NMEU09-1 and FJQEU14 isolates have smaller deletions (two-amino-acids) in the same region. Meanwhile, HK3, BJEU06-1, NMEU09-1, NVDC-FJ, LNEU12, FJEU13, FJQEU14, 15HEN1, HENZMD-10, KZ2018, and SC2020-1 isolates contain deletions in the same GP3 and GP4 overlap region as SD1291. The HU18755 isolate from Hungary in 2016 has the exact same five-amino-acid deletion in the GP3 and GP4 overlap regions as SD1291. Even though either the four-amino-acid deletion in nsp2 or the five-amino-acid deletion in the GP3 and GP4 overlap region have been identified in other PRRSV1 isolates, the concurrent deletions in SD1291 has not been identified before, suggesting the generation of a new pattern of deletion. In addition, the deletion or insertion of nsp2 might influence the viral immunogenicity and modulate the viral virulence [45, 46]. Furthermore, the GP3 and GP4 overlap region might be subjected to selective evolutionary pressure [47], the similar deletion patterns in this region not only suggested that these isolates might have the same or a closely related ancestor, but also indicated that these deletions might be beneficial for gaining fitness to escape from current PRRSV2 vaccination pressure in China. However, the exact correlation between these deletions/insertions and pathogenicity deserves further investigation.

Even though PRRSV1 isolates have been detected in the majority of the provinces of China, the pathogenicity of very few isolates was determined. The 2011 GZ11-G1 isolate shared the highest genome homology (96.3%) with the Amervac vaccine strain, which has been determined to be a virulent PRRSV1 isolate causing fever (∼40°C), high viremia, and mild clinical signs (anorexia, coughing, and sneezing) and lung lesions [19]. The 2014 HLJB1 isolate is a recombinant virus from a wild-type BJEU06-1 isolate and Amervac vaccine strain [24], which could cause fever, mild clinical signs (reddening of the conjunctiva, skin cyanosis, and dyspnea), and lymph node and spleen enlargement [48]. In this study, the 2022 SD1291 isolate is not recombinant and shares low similarity with other known PRRSV1 isolates, which could cause high fever (the highest is 41°C), common clinical symptoms (coughing, anorexia, and diarrhea) and mild lung consolidation. However, IHC did not identify PRRSV-specific antigen at 21 DPC, suggesting that SD1291 viral antigen could be rapidly eliminated by the host immune system. All these results indicated that the current PRRSV1 isolates circulating in China are pathogenic for piglets.

HLJB1 is another PRRSV1 previously isolated in our group [24]. As shown in Figure 5, both SD1291 and HLJB1 are grouped with BJEU06-1-like isolates. However, HLJB1 and SD1291 isolates share low genome similarity. In addition, both HLJB1 and SD1291 could only be isolated with PAMs but not with Marc-145 cells (Figure 1). To compare their replication efficacies, here, we determined the replication efficacies of HLJB1 and SD1291 in PAMs. The 2022 SD1291 isolate has higher replication efficacy than the 2014 HLJB1 isolate in PAMs, confirming that SD1291 has increased fitness for host target cells.

Even though the pathogenicity of Chinese subtype 1 PRRSV1 is currently low compared with the HP-PRRSV2 and NADC30-like PRRSV2 isolates, it cannot be guaranteed that any of the highly mutated PRRSV1 variants would become highly virulent. Actually, the Belgium 13V091 isolate has been identified as a highly pathogenic subtype 1 PRRSV1 strain, which could cause the highest respiratory disease scores and longest period of fever when compared with the other two PRRSV1 isolates (13V117 and 07V063) [49]. In addition, an Italian PR40/2014 isolate was also identified as a highly pathogenic subtype 1 PRRSV1 strain, which could result in severe clinical signs (high fever and dyspnea) and severe pathological lesions [50]. Therefore, the pathogenicity of novel subtype 1 PRRSV1 isolates should be evaluated regularly.

Although SD1291 is a relatively low-pathogenic isolate, the isolation of SD1291 from an adult slaughter pig suggests that the SD1291 isolate seems to have already broken through the current PRRSV immunization strategies using PRRSV2 vaccines. In another study, we inoculated pigs with the PRRSV1 HLJB1 isolate and obtained sera with neutralizing activity against HLJB1. However, these sera could not neutralize the SD1291 isolate (data not shown). This preliminary data supported the highly mutated SD1291 isolate could evade the protective immunity induced by other Chinese PRRSV1 isolates. No PRRSV1 vaccine is currently available in China. Therefore, more attention is required for monitoring the evolution and circulation of highly diverse PRRSV1 isolates in Chinese swine herds.

In conclusion, we first isolated a novel PRRSV1 strain from an adult slaughter pig in this study. The SD1291 shares low genome similarity with known PRRSV1 isolates and contains a new pattern of deletions in the nsp2 and GP3 and GP4 overlap regions. An animal challenge study showed that SD1291 is a pathogenic isolate for piglets. Our findings remind us that more attention is required to monitor the evolution and circulation of PRRSV1 in adult pigs in China.

## Figures and Tables

**Table 1:** Clinical sample information used for PRRSV1 detection.

Region (city, province)	Date	Sample no	PRRSV1 positive no	Percentage (%)
Linyi, Shandong	Feb. 24, 2022	31^*∗*^	1	3.23
Dezhou, Shandong	Feb. 26, 2022	30	0	0
Zhumadian, Henan	Feb. 25, 2022	27	0	0
Zhoukou, Henan	Feb. 25, 2022	39	0	0
Neijiang, Sichuan	Feb. 27, 2022	23	0	0
Hangzhou, Zhejiang	Feb. 24, 2022	53	0	0
Chaozhou, Guangdong	Feb. 27, 2022	31	0	0
Yangzhou, Jiangsu	Mar. 30, 2022	23	0	0
Total		257	1	0.39

^*∗*^The samples were collected from slaughter houses or farmer's markets.

**Table 2:** Detailed complete genome comparison of SD1291 to representative type 1 PRRSV strains.

Region	Length^*∗*^	*Similarity to SD1291 (%)*				
		LV	Amervac	BJEU06-1	HeB47	HLJB1
*Nucleotides (nt)*						
5′UTR	221	95.93	95.48	96.38	96.83	94.12
ORF1a	7176	85.58	83.94	86.22	86.08	82.92
ORF1b	4392	89.53	88.14	89.89	89.89	87.48
ORFs 2–7	3174	88.84	87.86	88.43	89.43	86.77
3′UTR	114	90.35	89.47	92.11	93.86	92.11
Complete	15068	87.59	86.19	88.09	88.12	85.28

*Proteins (aa)*						
Nsp1*α*	180	93.89	92.22	91.11	94.44	92.22
Nsp1*β*	205	80.98	79.02	87.80	86.83	84.88
Nsp2N	726	74.97	72.78	75.90	75.82	71.74
Nsp2TF	895	77.33	75.67	78.21	78.67	74.59
Nsp2	1073	80.89	79.22	82.29	81.44	77.93
Nsp3	230	93.48	93.48	93.48	93.48	93.48
Nsp4	203	91.63	91.13	92.61	92.61	90.64
Nsp5	170	92.35	92.35	93.53	92.94	93.53
Nsp6	16	100	100	100	100	100
Nsp7*α*	149	96.64	95.30	95.97	97.32	95.30
Nsp7*β*	107	91.59	91.59	92.52	94.39	93.46
Nsp8	45	91.11	88.89	93.33	91.11	88.89
Nsp9	685	96.64	96.50	96.35	96.50	96.06
Nsp10	442	95.48	95.25	96.38	95.70	95.02
Nsp11	224	96.88	96.88	97.77	97.77	96.88
Nsp12	152	92.11	93.42	91.45	92.76	92.11
GP2a	249	90.76	87.55	92.77	93.57	90.76
*E*	70	94.29	94.29	97.14	97.14	95.71
GP3	260	84.91	84.15	85.00	84.15	82.33
GP4	178	86.34	86.89	88.76	87.98	84.15
GP5	201	87.56	87.56	86.57	89.05	86.57
GP5a	43	95.35	93.02	88.37	95.35	95.35
*M*	173	95.38	94.80	91.33	94.80	93.64
*N*	128	92.19	92.19	91.41	93.75	89.06

^*∗*^The length of each fragment/protein in SD1291 genome. The cleavage products were determined based on previous description [26, 27, 41].

## Data Availability

The data used to support the findings of this study are included within the article.

## Supplementary Materials

Supplementary Materials Supplementary Table S1: Metagenomics sequencing quality.
