# Supplementary material for: Metagenomic and Pathogenic Assessments Identify a Pathogenic Porcine Reproductive and Respiratory Syndrome Virus 1 with New Deletions from Adult Slaughter Pig in 2022
Source: Transbound Emerg Dis. 2023 Apr 13;2023:1975039. doi: 10.1155/2023/1975039 (PMC12016735; doi:10.1155/2023/1975039)
Supplement: Supplementary Materials — Supplementary Table S1: Metagenomics sequencing quality. [file 1975039.f1.docx]

Table S1. Metagenomics sequencing quality.

| QC | Before filtering | After filtering |
| --- | --- | --- |
| Total_reads | 64806648 | 60603318 |
| Total_bases | 9785803848 | 8232789115 |
| Q20_bases | 9286816672 | 7981450615 |
| Q30_bases | 8782267575 | 7611675129 |
| Q20_rate | 94.90% | 96.95% |
| Q30_rate | 89.74% | 92.46% |
